# Selective glucocorticoid receptor-activating adjuvant therapy in cancer treatments

**DOI:** 10.18632/oncoscience.315

**Published:** 2016-07-27

**Authors:** Nora Sundahl, Dorien Clarisse, Marc Bracke, Fritz Offner, Wim Vanden Berghe, Ilse M. Beck

**Affiliations:** ^1^ Laboratory of Experimental Cancer Research (LECR), Department of Radiation Oncology & Experimental Cancer Research, Ghent University, Gent, Belgium; ^2^ Cancer Research Institute Ghent (CRIG), Ghent, Belgium; ^3^ Receptor Research Laboratories, Nuclear Receptor Lab (NRL), VIB Medical Biotechnology Center, Ghent University, Ghent, Belgium; ^4^ Hematology, Department of Internal Medicine, Ghent University, Ghent, Belgium; ^5^ Laboratory of Protein Chemistry, Proteomics and Epigenetic Signaling, Department of Biomedical Sciences, University of Antwerp, Wilrijk, Belgium

**Keywords:** glucocorticoids, selective glucocorticoid receptor agonist, selective glucocorticoid receptor modulator, cancer, hematological malignancies, therapy resistance

## Abstract

Although adverse effects and glucocorticoid resistance cripple their chronic use, glucocorticoids form the mainstay therapy for acute and chronic inflammatory disorders, and play an important role in treatment protocols of both lymphoid malignancies and as adjuvant to stimulate therapy tolerability in various solid tumors. Glucocorticoid binding to their designate glucocorticoid receptor (GR), sets off a plethora of cell-specific events including therapeutically desirable effects, such as cell death, as well as undesirable effects, including chemotherapy resistance, systemic side effects and glucocorticoid resistance. In this context, selective GR agonists and modulators (SEGRAMs) with a more restricted GR activity profile have been developed, holding promise for further clinical development in anti-inflammatory and potentially in cancer therapies. Thus far, the research into the prospective benefits of selective GR modulators in cancer therapy limped behind. Our review discusses how selective GR agonists and modulators could improve the therapy regimens for lymphoid malignancies, prostate or breast cancer. We summarize our current knowledge and look forward to where the field should move to in the future. Altogether, our review clarifies novel therapeutic perspectives in cancer modulation via selective GR targeting.

## INTRODUCTION

Therapeutic administration of glucocorticoids (GCs) is frequently applied to combat inflammatory, immune and allergic disorders, brain edema, shock and various blood cancers [1–4]. However, in malignancies the effect of GCs is ambiguous; it has an anti-cancer effect in most hematological malignancies [5–8], yet in solid tumors GCs have been shown to inhibit as well as promote tumor progression [9–21]. Recent advances in the development of selective glucocorticoid receptor agonists and modulators (SEGRAMs) have yielded several compounds which bind to GR yet exert a different, more specific, action radius as compared to classic GCs [3, 22–30]. In the light of this development, some of these SEGRAMs have also been studied in the context of malignancies [27, 30–[45]. In this article we will review what is currently known about the effect of SEGRAMs on the induction and progression of cancer.

## GLUCOCORTICOIDS AND GLUCOCORTICOID RECEPTOR FUNCTION

**Figure 1:**
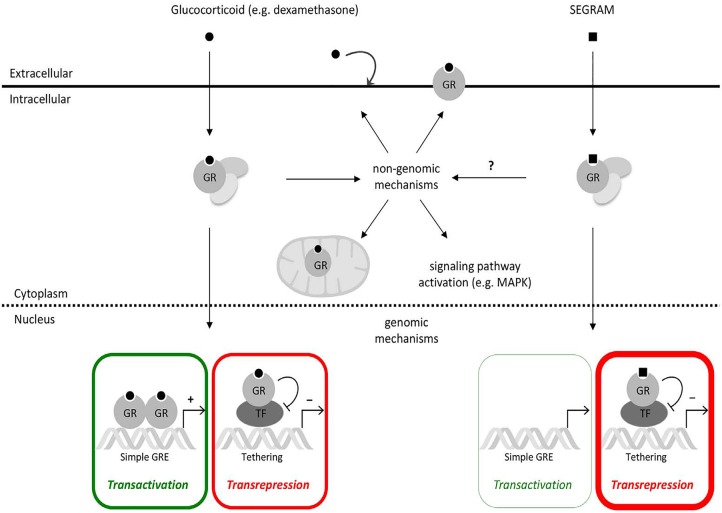
Genomic and non-genomic mechanisms of GR-mediated signaling Classical GCs such as dexamethasone can enter the cell via passive diffusion and bind to GR. Upon GC binding, GR is released from the multiprotein complex and can execute either genomic or non-genomic mechanisms. The genomic mechanisms include transactivation, promoting transcription of target genes, and transrepression, inhibiting transcription factor (TF)-driven gene expression. The non-genomic mechanisms consist of GCs modulating cell membranes, membrane-bound GR, cytosolic and membrane-bound GR modulating signaling pathways and GR translocation into mitochondria. The SEGRAM-driven GR can, similar to classical GCs, translocate to the nucleus and repress TF-driven (NF-κB- and/or AP-1) driven gene expression, but not (or to a lesser extent) result in the upregulation of GRE-mediated genes such as GILZ. How these SEGRAMs affect non-genomic GR-mediated mechanisms such as kinase activities is unknown.

In its unliganded state, the glucocorticoid receptor (GR) is a predominately cytoplasmic protein and its ligand-binding conformation is maintained by its chaperoning partners in a large multi-protein complex. Nonetheless, GR is able to shuttle between the nucleus and the cytoplasm [46, 47]. The ligand-bound GR is subject to a conformational change and subsequently dissociates from the chaperoning complex. Ligand-bound GR is guided to the nucleus where it can fuel or inhibit the expression of specific genes via different mechanisms, including binding to glucocorticoid-responsive elements (GREs) in the genes’ promoter regions and interacting with other DNA-bound transcription factors, as such influencing gene expression (Figure 1) [48]. Thus, GCs stimulate the expression of anti-inflammatory proteins, metabolic gene products and certain pro-apoptotic proteins [48–51]. They can also inhibit the transcription of pro-inflammatory proteins (e.g. cytokines, enzymes and adhesion molecules) and certain anti-apoptotic proteins through a number of different mechanisms [49–52]. Besides these GR-mediated genomic mechanisms, non-genomic GR-mediated mechanisms have been described involving membrane-modulating effects of GCs, modulation of signaling pathways by membrane- associated GRs, cytosolic GRs and mitochondrial GRs [53–58].

## CANCER MODULATION VIA DIRECT OR INDIRECT EFFECTS OF GLUCOCORTICOIDS

GCs have a variety of cell-specific effects on cancer cells (Table 1). In hematological malignancies GCs can have anti-cancer effects [7, 50, [59]. However, in solid tumors the *in vitro* and *in vivo* data are controversial. On the one hand, it has been shown that GCs inhibit apoptosis and prevent chemotherapy-induced apoptosis in most solid malignancies [9–12, 16, 17], thereby stimulating tumor progression as indicated by an increased likelihood of metastasis in breast cancer patients [13, 14] or increased risks of skin cancer among users of systemic GCs [15]. Also an elevated risk of non-hodgkin lymphoma was seen among these users of systemic GCs [15]. These results were confirmed by *in vitro* and *in vivo* data obtained with various human carcinoma cell lines and mouse tumor xenografts [12, 16]. On the other hand, GCs have also been identified as a chemosensitizer [10, 60, [61]. Similarly, in prostate cancer GCs can either inhibit tumor growth or induce chemotherapy resistance [18–21]. Nonetheless, GCs have been used in cancer therapy since the 1940s. Nowadays, they are commonly found in the regimens of acute lymphocytic leukemia, chronic lymphocytic leukemia, Hodgkin's and non-Hodgkin's lymphoma, multiple myeloma [7, 8] and prostate cancer [18]. Furthermore, they are used as adjuvant therapy in the treatments of various solid tumors to avoid excessive immune reactions and healthy cell toxicity in response to chemotherapy, to reduce nausea and emesis, to decrease swelling, to inhibit tissue reaction (e.g. inflammation) against invasive tumor growth or as palliative treatment of metastasis-related pain [8]. The cancer-modulating mechanisms of GCs are not yet fully understood, but a variety of such mechanisms has been postulated and investigated [8, 18, 19, 59].

**Table 1:** The effect of glucocorticoids on different tumor entities

Malignancy	Effects of GCs	Refs
Hematological malignancies	Induction of apoptosis via upregulation of pro-apoptotic genes, transrepression of NF-κB and AP-1 and non-genomic mechanisms.	[5, 6, 63, 64, 67–71]
Breast cancer	Depending on cell type and GR and ER status, inhibition of chemotherapy-induced apoptosis via inactivation of MAPKs, transrepression of certain genes and transactivation of DUSP1/MKP1 and SGK-1.	[9, 76–79, 155]
Skin tumor	Prevention of skin tumor promotion. Resistance to GCs in established papillomas and carcinomas.	[81–89]
Prostate cancer	Progression inhibition in hormone-refractory prostate cancer via inhibition of adrenal androgen production and the modulation of gene expression. Progression stimulation in tumors receiving anti- androgen therapy via diminishing the efficacy of anti-androgen therapy and transactivation of pro-cell survival genes.	[18, 19, 90–95]

In hematological malignancies, the major effect of GCs is induction of apoptosis of cancer cells [5, 6]. The exact mechanism has not yet completely been clarified. Nevertheless, intervals of continuously administered steroids, e.g. dexamethasone, and sufficient levels of functionally receptive and active GR in malignant lymphoid cells are essential to reach therapy effectiveness. This evokes a culmination of genomic, including transactivation and transrepression, and non- genomic regulatory events, suggesting the involvement of a complex crosstalk between GCs and diverse other signaling pathways [5]. GR transactivation is required for apoptosis via upregulating pro-apoptotic genes, such as Bim and suppressor of AP-1 regulated by interferon (SARI) [6, 62–[65]. Other studies stress the importance of GC-induced transrepression of the nuclear factor κB (NF- κB) and activator protein 1 (AP-1) as a driver of apoptosis, impairing transcription of pro-inflammatory cytokines, such as IL-6, anti-apoptotic genes, such as Bcl-xL, and cell cycle promoting genes, such as cyclin D1 [50, 51, 62, 66]. Non-genomic mechanisms such as translocation of GR into mitochondria were shown to reduce the mitochondrial outer membrane potential, promoting the release of cytochrome C, a prerequisite for the activation of the intrinsic apoptotic pathway [56, 67]. Until today, combination of traditional chemotherapies and newer agents (such as proteasomal inhibitors) with GCs remain a cornerstone in the treatment of lymphoid malignancies, especially for multiple myeloma and acute lymphoblastic leukemia therapy [68–71].

During the treatment of breast cancer, GCs are often administrated as an adjuvant therapy to reduce the adverse effects of chemotherapy and to protect the normal tissue against the long-term effects of genotoxic drugs [72]. Yet, in 1958, an autopsy study on patients with disseminated breast carcinoma showed that 26% (8 of 31) of women who received glucocorticoid treatment developed spleen metastases, whereas women who had not received the therapy, did not develop spleen metastases (0 of 16) [11, 13, [73]. This was confirmed by a following autopsy study on gastroduodenal metastases, where 29 out of 136 patients (21%) who had received corticosteroids developed metastases to the gastroduodenal area as compared to 9 out 68 patients (13%) of the control group [73, 74]. GCs can inhibit apoptosis in breast cancer cells *in vitro* and *in vivo* [75]. More specifically, they inhibit chemotherapy- induced apoptosis, via GR-mediated inactivation of mitogen-activated protein kinases (MAPKs), GR-mediated inhibition of certain genes, including Insulin-like growth factor-binding protein 3 (IGFBP-3) and tissue plasminogen activator and GR-fueled increased expression of dual- specificity phosphatase 1 (DUSP1)/mitogen-activated protein kinase phosphatase-1 (MKP1) and serum/glucocorticoid regulated kinase 1 (SGK-1) [10, 76–[79]. The latter mechanism suggests that transrepressive GC compounds lacking transactivation may remain effective while reducing adverse effects. Regardless of the negative impact of exogenous GC administration, GR expression itself is not a reliable prognostic factor for tumor size, stage and grade. Moreover, sustainable expression of GR has been associated with several beneficial outcome features, among which estrogen receptor, progesteron receptor, Forkhead Box A1, GATA -binding protein 3 and brain-expressed X-linked 1 (BEX1) expression [80].

In skin tumor models, induced by chrysarobin, 7-bromomethylbenz[a]anthracene (BrMBA) or 12-O-tetradecanoylphorbol-13-acetate (TPA), GCs can prevent skin tumor promotion *in vitro* and *in vivo* [81–86]. Furthermore, transgenic animals expressing high levels of GR in the skin seemed to be highly resistant to skin tumor development [83, 87]. However, several tumorigenic keratinocyte cell lines appeared functionally GC-resistant to GC-induced growth arrest, regardless of the high levels of GR mRNA and protein [88]. Moreover, established papillomas and carcinomas appear to be resistant to GCs [88, 89].

The role of GR in prostate cancer is rather ambiguous. On the one hand, GCs are often a part of complex chemotherapy in advanced hormone-refractory prostate cancer. Their anti-cancer effects are attributed primarily to their inhibitory effect on adrenal androgen production. Recent evidence shows that GCs also directly target prostate cancer cells through modulation of the expression of genes regulating growth, apoptosis, inflammation, metastasis, differentiation, cell survival and angiogenesis [18, 19, 90–93]. On the other hand, prostate tumors that have received prolonged androgen receptor (AR)-blocking anti-androgen therapy (e.g. enzalutamide) display a relatively higher GR expression level which rather increases cell viability and facilitates progression in vivo [94, 95]. GR-mediated actions also diminish the efficacy of AR inhibition therapy and even stimulate the expression of pro-cell survival genes. In some prostate cancers anti-androgen resistance has been linked to upregulation of GR. Since GR and AR have partially overlapping target genes, upregulation of GR may restore the transcription of one or more AR target genes following AR inhibition therapy [94]. This puts forth the idea that whereas GR activation has a detrimental effect on AR signaling-deficient prostate cancer, it can also inhibit the progression of prostate cancers from androgen dependence to hormone resistance as long as AR is still functionally active.

The effect of GCs on the efficiency of chemotherapy and radiotherapy is controversial. In several established cell lines as well as primary carcinoma cells from solid tumors (i.e. bone, brain, breast, cervix, melanoma and neuroblastoma) GCs could prevent chemotherapy-induced cell death and even promote proliferation [12, 75, 96–110]. Also in the clinical setting, it has been observed that concomitant combination chemotherapy with GCs resulted in a worse outcome than single chemotherapy [111, 112]. In radiotherapy mouse models, GCs could have an indirect positive effect on radiosensitivity via decreasing tumor oxygen consumption, and thereby enhancing tumor oxygenation [113]. Paradoxically, GC-induced resistance to radiotherapy [114] and GR antagonist-enhanced radiosensitivity have also been reported [115, 116].

## SELECTIVE GLUCOCORTICOID RECEPTOR AGONISTS AND MODULATORS (SEGRAMS)

**Figure 2:**
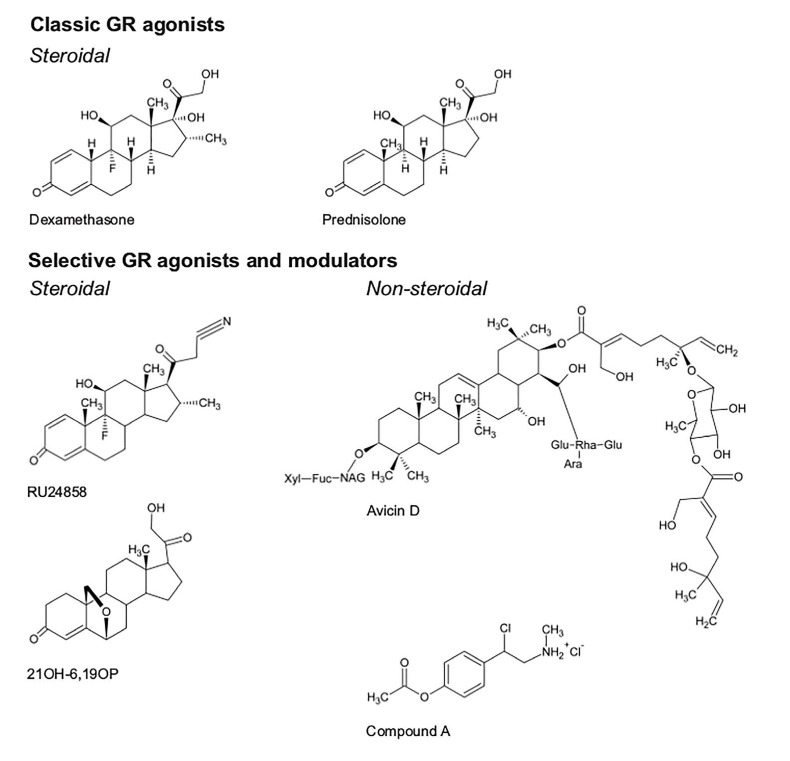
Chemical structures of discussed GR-targeting compounds Classical GR agonists such as dexamethasone and prednisolone have a steroidal skeleton. Selective GR agonists and modulators are divided in two categories: steroidal, RU24858 and 21OH- 6,19OP, and non-steroidal, avicin D and compound A, compounds.

Selective glucocorticoid receptor agonists and modulators (SEGRAMs) are a class of compounds which act on GR in analogy to GC, yet affect GR-mediated gene expression in a different manner, skewing the bulk of gene expression modulation to either transrepression or transactivation, but with the intention to improve the therapeutic efficacy. Although in inflammatory afflictions, a new model balancing transrepression and transactivation more carefully is now appearing [3, 22, [23], a few decades ago the key anti-inflammatory effect of GR was attributed to its transrepression mechanism [117–119], while the majority of GC-associated side effects was originally ascribed to GR transactivation [120]. This pharmacological model launched a series of compounds exerting primarily transrepressive effects of GR for treatment of inflammatory disorders [3]. As such, RU24858 [24], 21OH-6,19OP [25, 26], avicin D [27], compound A (CpdA) [28], (Figure 2) and many more [3, 29] have been classified as SEGRAMs.

How shifting the balance to a relative preponderance in GR transactivation or GR transrepression affects therapeutic efficacy related to tumor development, tumor growth, tumor cell apoptosis or GC/GR-induced chemotherapy resistance is not completely clear yet. But since it is known that GR can affect malignancies in a stimulating and inhibiting manner (as discussed above), increased interest into whether SEGRAMs can skew the action radius of GR to solely tumor suppression spikes in literature.

Evidence that GR transrepression and GR transactivation can be dissociated originates from a pioneering study with a GR dimerization mutant (GRdim) [117]. This mutant harboring a point mutation in the second zinc finger of the GR DNA-binding domain, has difficulties forming GR homodimers, which abrogates high affinity binding to GRE motifs at specific gene promoters. This abolishes to a large extent GR transactivation effects whereas transrepression capacity remains largely unaffected [121, 122]. In multiple myeloma, *GRdim*-based experiments reveal a specific role for classic GR transactivation in GR-mediated apoptosis [123].

The well-known GR antagonist RU486 (also known as mifepristone), is incorporated into various cancer regimens because of its antineoplastic potential. However, RU486 is also a progesterone and androgen receptor antagonist, and most research attributes the antineoplastic effect to the antagonism of the progesterone receptor [124]. Yet, current findings question this hypothesis [125]. Since RU486 has an antagonistic effect and is not GR specific, it is not considered a SEGRAM.

Our review will particularly focus on RU24858, 21OH-6,19OP, Avicin D and CpdA, as these are the only SEGRAMs, for which potential cancer-modulating properties have been reported so far.

The underlying mechanism for differential GR regulation by SEGRAMs and GCs has not completely been resolved. CpdA [28], avicin D [27], RU24858 [126] and 21OH-6,19OP [127] can instigate a GR translocation to the nucleus in analogy to classic GCs (e.g. dexamethasone), although potencies may vary [38, 128–130]. Research indicates that CpdA [28], RU24858 [126] and 21OH-6,19OP [131] induce a different conformational change in GR than GCs do. *In silico* modeling maps CpdA [38], RU24858 [126] and 21OH-6,19OP [132] in the ligand-binding pocket of GR; whether this is also the case for avicin D is still unknown. However, even though the virtual docking studies show that CpdA could bind in the ligand-binding pocket, additional structural studies are still required. While CpdA promotes a monomeric GR conformation [127, 130], the compound 21OH-6,19OP [25] drives GR to a dimeric formation, just as GCs do. It still needs to be investigated whether RU24858 and avicin D favor a predominately monomeric or dimeric GR conformation. The binding affinity to GR of the discussed SEGRAMs can be found in Table 2.

**Table 2:** Binding affinity of discussed SEGRAMs to GR

SEGRAM-mediated binding to GR			
SEGRAM	GR, origin	GR binding	Ref
RU24858	A549 cells, h	Ki = 110.0 ± 24.0 nM	[134]
21OH-6,19OP	rat thymus	Kd = 125 nM (RBA for GR as compared to corticosterone)	[26]
Avicin D	A549 cells, h	competition for Dex 35% (with 5-fold excess); 85% (with 500-fold excess)	[27]
CpdA	L929sA, m	IC50 = 6.4 nM (CI 1.9-20.5 nM)	[28]
	BWTG3, m	Kd = 81.8 nM	[156]
	COS-1, s, TT hGR	IC50 = 0.003 ± 0.004 nM	[157]
	DU145 cells, h	competition for Dex 85% (with 100-fold molar excess)	[38]
**Dexamethasone**	A549 cells, h	Ki = 4,9 ± 1.3 nM	[134]
	L929sA, m	IC50 = 25.9 nM (CI 7.9-84 nM)	[28]
	BWTG3, m	Kd = 1.29 nM	[156]
	COS-1, s, TT hGR	IC50 = 14 ± 4 nM	[157]

Abbreviations: RBA, relative binding affinity; CI, confidence interval; h, human; m, murine; s, simian; TT, transiently transfected; IC50, concentration at which compound inhibited 50% of specific binding of labeled dexamethasone.

Similarly to classical GCs, CpdA [133], avicin D [27], 21OH-6,19OP [25, 26], and RU24858 [134], efficiently downregulate the transcription of TNF-induced and NF-κB-dependent pro-inflammatory genes (e.g., IL-6, IL-8, E-selectin) (Figure 1) [28].

In contrast to classical GCs, CpdA [28], avicin D [27] and 21OH-6,19OP [132] do not stimulate endogenous GRE-dependent genes. This means that these compounds only exhibit transrepression effects, but lack transactivation capacity (Table 3) [27, 28]. This is in contrast to RU24858 [126] which still induces GR transactivation in selected cell types [135]. In mouse models, in contrast to dexamethasone, CpdA shows anti- inflammatory effects and displays reduced GC side-effects because it does not induce hyperglycemia (potentially leading to diabetes) [28, 136], hyperinsulinemia [130] or skin atrophy [137] and does not elevate AST and ALT enzyme levels (which is a sign of liver toxicity) [138]. Endogenous cortisol levels are also maintained by CpdA [138]. Finally, whereas prolonged GC treatment frequently downregulates GR levels and contributes to the onset of GC resistance, prolonged CpdA treatment does not decrease GR levels, allowing sustainable anti-inflammatory effects [122, 128, [139]. Research investigating the *in vivo* adverse effects of the SEGRAMs RU24858, 21OH-6,19OP and avicin D is yet to be performed. How the SEGRAMs RU24858, 21OH-6,19OP, avicin D and CpdA affect the non-genomic effects of GR is currently still largely unknown.

## SEGRAMS’ CANCER-MODULATING POTENTIAL

**Figure 3:**
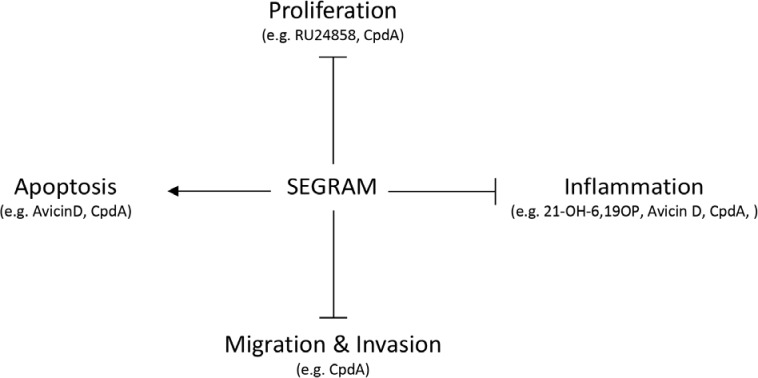
SEGRAM-triggered mechanisms in cancer SEGRAMs have anti-inflammatory properties, can inhibit proliferation, migration and invasion, and can induce apoptosis of cancer cells. However, the specific action mechanism depends on the SEGRAM itself and on the tissue type.

Although the SEGRAMs discussed below are not the only SEGRAMs known to literature [3, 29], they are the only ones to our knowledge that have been evaluated for their cancer-modulating potential (Figure 3).

### RU24858

It has been revealed by competition assays *in vitro* that the SEGRA RU24858 binds GR with similar affinity as dexamethasone [24]. Since GCs can prevent skin cancer formation elicited by specific chemicals or ultraviolet radiation in animal models [82, 140, [141], the dissociated compounds RU24858 and RU24782 have been investigated in this setting. In a SENsitivity to CARcinogenesis (SENCAR) mouse model classical GCs and the compound RU24858 were both able to prevent epidermal hyperplasia and proliferation elicited by exposure to tumor-promoting TPA. The anti-proliferative and anti-hyperplastic effects of RU24858 in these mice were weaker than that of the classic GCs. Furthermore, RU24858 was also able to reverse the induction of TPA-induced genes, associated in skin tumor promotion (such as c-Jun, COX-2 and iNOS) [31]. In contrast, the comparable compound RU24782, which has a thioether functional group instead of a nitrile functional group as compared to RU24858, failed to elicit anti-cancer activities [31]. The different behavior of these SEGRAs could be due to a varying inhibition of NF-κB and/or a varying ability to interact with repressive histone deacetylases [31, 142–[144].

### 21OH-6,19OP

21OH-6,19OP is a selective GR agonist, which lacks the bulky substituent at C-11, found in active GR antagonists [26, 145]. This compound is able to transrepress AP-1 and NF-κB pathways in COS-1, RAW 264.7 and BHK cells, yet not in A549 lung cancer cells [25, 37, [132]. In the latter cells, 21OH-6,19OP was shown to block TNF-induced COX2 and IL-8 expression [37]. Furthermore, it is largely incapable of inducing transcription of GRE-regulated genes [25, 26] and, when incubated in combination with dexamethasone, it acts as an antagonist to GR transactivation [26]. These findings indicate that 21OH-6,19OP is a cell-specific, selective GR agonist. Noteworthy, 21OH-6,19OP is capable of upregulating DUSP1, most likely contributing to its cytokine-inhibiting capacities [37].

As it is known that GCs, administered for symptom- reducing reasons, can induce chemoresistance in certain malignancies [11], SEGRAM-based research could shed light on the mechanistic 21OH-6,19OP basis of this phenomenon. 21OH-6,19OP could circumvent this mechanism as *in vitro* and *in vivo* research indicates that contrary to the classic GC dexamethasone, 21OH-6,19OP does not preclude cell death triggered by the anthracyclin doxorubicin in mammary tumor cells. Concomitantly, 21OH-6,19OP does not avert paclitaxel-stimulated caspase-3 activation and does not stimulate anti-apoptotic BCL-X_L_ isoform gene expression [37].

### Avicin D

Avicin D [30] is a desert plant-derived triterpenoid saponin [32], with GR-mediated anti-inflammatory effects [27]. It has also been shown that avicin D induces apoptosis in a variety of human tumor cell lines, such as T-cell leukemia cells (Jurkat cells), breast cancer cells (MDA-MB-435), prostate cancer cells (PC3) and cutaneous T-cell lymphoma cells (CTCL) [27, 30, 32–36]. Contrary to its anti-inflammatory effects, current research indicates that avicin D's cytotoxicity is GR-independent [27]. The underlying mechanism of apoptosis induction is most likely a direct effect of avicin D on mitochondrial bioenergetics which subsequently triggers the apoptosis cascade [33, 146, [147].

### Compound A

Compound A is a desert plant-derived SEGRM that exhibits GR transrepression capacities, and lacks the ability to fuel classic GR transactivation. In malignant prostate, bladder, leukemia and multiple myeloma cell lines and primary leukemia cells, CpdA can induce apoptosis [38–42]. Contrary to avicin D, its pro-apoptotic and cytotoxic effects are reported to be partially GR- dependent [38, 39, 41, 42].

In advanced prostate cancer, activation of the AR has a pro-tumorigenic effect, whereas exclusive activation of GR can have an anti-tumorigenic effect [18, 19, 90–93]. The SEGRM CpdA can affect both of these steroid receptors in different cell lines, among which prostate cancer cells [38, 43]. Virtual docking studies showed that CpdA was able to bind the AR. However, CpdA was not able to compete for binding to the AR, in contrast to the androgen mibolerone. Nevertheless, CpdA induces the nuclear translocation of AR, but inhibited AR DNA binding and AR transcriptional activity [38, 43]. Thus, the effect of CpdA resembles the effect of anti-androgens, commonly used in the therapy of prostate cancer [43]. CpdA shifts the activity of GR towards transrepression, theoretically resulting in the inhibition of pro- proliferative and anti-apoptotic gene expression. However, experimental evidence to substantiate this is currently lacking. Remarkably, CpdA seems able to upregulate the protein levels of pro-apoptotic Bim and tumor suppressor p53 in both CEM and K562 leukemia cells [40].

CpdA's selective GR actions and to a lesser extent its antagonistic AR actions also contribute to the compound's ability to inhibit bladder cancer cell proliferation, colony formation, cell migration and invasion, and to increase cell cycle arrest and apoptosis [41]. The combination of AR blockage and GR-mediated transrepression resulted in an inhibition of prostate tumor growth *in vitro* [38] and bladder cancer cell xenograft growth *in vivo* [41]. Furthermore, CpdA induced increased caspase activation and PARP cleavage in these prostate cancer cells [38].

Currently, the proteasomal inhibitor bortezomib is often used in therapy for lymphomas and myelomas [148]. The combination of bortezomib and GCs has been proven to act synergistic in hemoblastoses [149]. This synergistic effect may be explained in several different ways. The proteasome inhibitor prevents GR cleavage, consequently GR levels rise and GR activity is reinforced. In addition, GCs enhance the expression of proteins implicated in the development of ER stress [40], and excessive ER stress induces apoptosis and sensitizes cells to bortezomib- induced apoptosis [150]. CpdA treatment had a more prominent cytostatic and apoptotic effect on leukemia cells as compared to treatment with the GC fluocinolone acetonide. Furthermore, the combined treatment with bortezomib and CpdA showed that CpdA potentiated the cytotoxic effect of bortezomib [40].

In breast cancer, Chen *et al*. recently discovered that dexamethasone, but not CpdA treatment clearly protects triple negative breast cancer cells (MDA-MB-231) from paclitaxel-induced growth inhibition [44]. Moreover, Chip-exo-based studies confirm that Dex-liganded, but not CpdA-liganded, GR can bind to a single GRE, which drives the expression of pro-tumorigenic genes. From the transrepression perspective, AP-1 and NF-κB-enriched motifs in triple negative breast cancer cells do not attract a tethering CpdA-liganded GR. Actually CpdA alters the expression of only a small number of genes that are not involved in carcinogenesis and therapy resistance [44]. These findings suggest that tweaking the action radius of GR may lead to a safer coadjuvant for chemotherapy for breast cancer.

In contrast to classic GCs and RU24858 [31], CpdA was not able to inhibit TPA-induced skin epidermal thickening and proliferation. Moreover, it even increased, yet to a lesser extent than TPA, the epidermal thickness and proliferation of keratinocytes in an *in vivo* model of SENCAR mice [45]. This effect can possibly be explained by the fact that CpdA-bound GR does not transrepress AP-1-regulated genes in particular cell types, and as such may be less effective in suppressing transcription of tumorigenesis-associated genes c-Jun, COX-2, and IL-6 [133]. Yet, CpdA was able to decrease the expression of the p50 subunit of NF-κB in these SENCAR mice. Of special note, since NF-κB is constitutively activated in 7,12-dimethylbenz[a]anthracene (DMBA)/TPA- promoted mouse skin tumors, partially due to an increase in expression/activation of p50, this pathway could have an interesting anti-tumoral potential [45].

Furthermore, the effect of CpdA was recently also investigated on cancer-surrounding cells, namely colon cancer-derived myofibroblasts, which influence cancer cells via cell-to-cell or paracrine signaling. This research revealed that in comparison to dexamethasone, CpdA only had minor impact on gene expression and protein levels of cancer-promoting factors [128].

## DISCUSSION

Although the cell type-specific anti-cancer potential of GCs involves inhibition of growth and induction of apoptosis, the mechanism by which GCs are able to do this has not been completely elucidated. Further research with currently available anti-inflammatory SEGRAMs, under clinical evaluation [3], will allow to dissect the mechanism of action of GR in neoplastic tissue. This will entail the design of SEGRAMs with very specific mechanisms of action to fight against cancer.

Despite its widespread use, chronic exposure to GCs in cancer treatments is severely limited by the vast range of side effects it induces or the development of therapy resistance [2]. Therefore, the development of clinically more selective compounds that exert the activation or inhibition of a subset of pathways may result in a more focused anti-cancer action, reduced risk of therapy resistance, but also a significant reduction in side effects and thus more comfort for the patient. Along the same line, the development of selective estrogen receptor modulators and selective androgen receptor modulators has yielded compounds with improved anti-cancer action and reduced side effects. Their selective action radius has allowed these compounds to be used also in primary prevention regimens against cancer (e.g. tamoxifen in breast cancer) [151], besides their classical use in integral cancer therapy, like chemotherapeutic agents such as cisplatin or paclitaxel. Spatiotemporal delivery restrictions could aid to confine the compounds’ actions even further and thus limit the side effect profile and increase its efficiency. The development of a liver-specific GR antagonist A-348441 (now called KB3305) with a clinical proof-of-concept shows that it is feasible to develop tissue- or organ-specific GR-targeting compounds [152]. In the framework of solid tumors and the associated leaky vasculature, recent work in metastatic prostate cancer bone lesions shows that liposomal encapsulated dexamethasone is delivered to these malignant bone lesions and sometimes inhibits *in vivo* tumor growth more efficiently than systemic administration of dexamethasone [153]. Also the recent research proving the anti-cancer effect of cationic lipid- conjugated hydrocortisone, which selectively targets cancer cells endorses the possibility of cell-type specific GR-targeting compounds [154].

In conclusion, combining selective GR modulator compounds, with innovative tissue- or cell- specific delivery methods may allow to further optimize therapeutic efficacy and reduce adverse GC-associated side effects. Further investigation of selective modes of action of GR should allow further development of GR dependent anti-cancer compounds for various malignancies, with improved specificity and optimized therapeutic window.
